# Supplementary material for: Novel Cyclophilin Inhibitor Decreases Cell Proliferation and Tumor Growth in Models of Hepatocellular Carcinoma
Source: Cancers (Basel). 2021 Jun 18;13(12):3041. doi: 10.3390/cancers13123041 (PMC8234462; doi:10.3390/cancers13123041)
Supplement: Supplementary file 1 [file cancers-13-03041-s001.zip › cancers-1253865-supplementary.pdf]

A

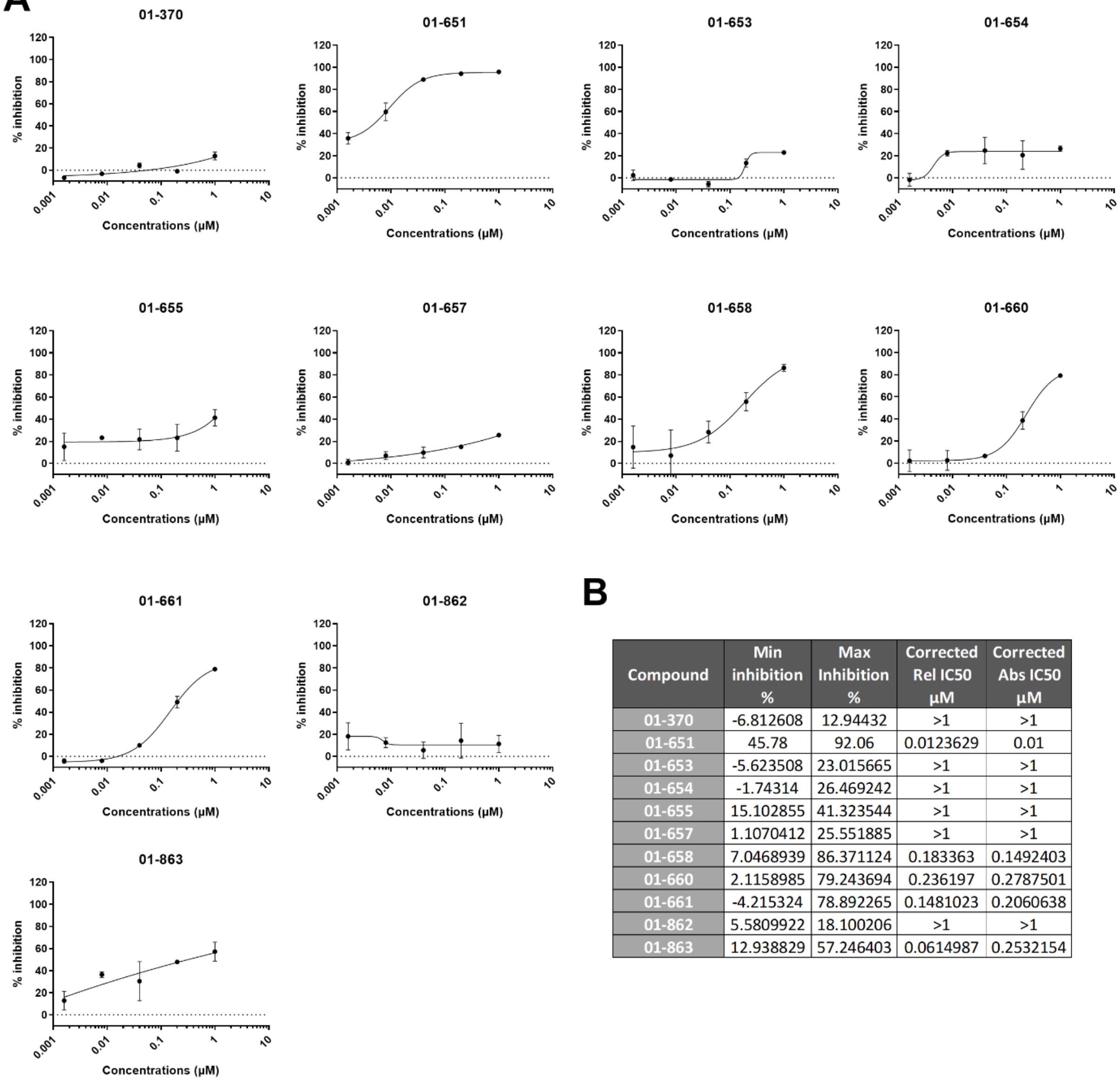


B

| Compound | Min inhibition % | Max Inhibition % | Corrected Rel IC50 μM | Corrected Abs IC50 μM |
|---|---|---|---|---|
| 01-370 | -6.812608 | 12.94432 | >1 | >1 |
| 01-651 | 45.78 | 92.06 | 0.0123629 | 0.01 |
| 01-653 | -5.623508 | 23.015665 | >1 | >1 |
| 01-654 | -1.74314 | 26.469242 | >1 | >1 |
| 01-655 | 15.102855 | 41.323544 | >1 | >1 |
| 01-657 | 1.1070412 | 25.551885 | >1 | >1 |
| 01-658 | 7.0468939 | 86.371124 | 0.183363 | 0.1492403 |
| 01-660 | 2.1158985 | 79.243694 | 0.236197 | 0.2787501 |
| 01-661 | -4.215324 | 78.892265 | 0.1481023 | 0.2060638 |
| 01-862 | 5.5809922 | 18.100206 | >1 | >1 |
| 01-863 | 12.938829 | 57.246403 | 0.0614987 | 0.2532154 |

**Figure S1.** Compound screening for SfA-derived cyclophilin inhibitors **(A)** Antiproliferative activity was evaluated in duplicates in a set of SfA-derived inhibitors at different doses in HUH-7 cells. Data are presented as Mean ± SD; **(B)** Table indicating the minimal and maximal inhibition and relative and absolute $IC_{50}$ values for the tested SfA-derived cyclophilin inhibitors
